# Ribosomal protein L9 is a potential therapeutic target for B-ALL through the activation of the p53 signaling pathway

**DOI:** 10.3389/fimmu.2025.1560706

**Published:** 2025-03-27

**Authors:** Xinxin Li, Wenting Meng, Xi Wang, Siyong Huang, Jianbin Wang, Han Liang, Dailing Si

**Affiliations:** ^1^ Xi’an Key Laboratory of Stem Cell and Regenerative Medicine, Institute of Medical Research, Northwestern Polytechnical University, Xi’an, China; ^2^ Research & Development Institute of Northwestern Polytechnical University in Shenzhen, Shenzhen, China; ^3^ Department of Neurosurgery, Xijing Hospital, Fourth Military Medical University, Xi’an, China; ^4^ Department of Hematology, Xi’an International Medical Center Hospital, Xi’an, China; ^5^ Department of Medical Genetics and Developmental Biology, Fourth Military Medical University, Xi’an, China; ^6^ Analysis & Testing Laboratory for Life Sciences and Medicine, Fourth Military Medical University, Xi’an, China

**Keywords:** B-ALL, RPL9, cell proliferation, apoptosis, p53 signaling pathway

## Abstract

B-cell acute lymphocytic leukemia (B-ALL) is a malignant hematological disorder marked by the aberrant proliferation of abnormal B lymphocytes. Although recent advancements have highlighted the pivotal role of ribosomes in the progression of B-ALL, the specific function of ribosomal protein L9 (RPL9), a key component of ribosomal structural protein, still unclear. In this study, we observed a significant upregulation of RPL9 in human B-ALL cells compared to normal B cells, suggesting RPL9’s potential key role in B-ALL progression. Enforced RPL9 knockdown (KD) led to decreased proliferation and increased apoptosis in B-ALL cells compared to the control group. Furthermore, RPL9 KD significantly extended the survival time of NCG mice bearing B-ALL cells *in vivo* compared to controls. Mechanistically, our findings indicate that RPL9 KD triggers nucleolar stress, disrupts ribosome biosynthesis, and activates the p53 signaling pathway. Building upon our recent investigation into the positive regulatory influence of FTO on m^6^A-modified RPL9, we discovered that FTO overexpression can mitigate the activation of p53 signaling induced by RPL9 KD. Our findings further suggest that RPL9 KD increases MICA/B mRNA and protein expression in B-ALL cells, which serves as crucial ligands of NK cell’s NKG2D, potentially heightening their sensitivity to NK cell-mediated cytotoxicity. In summary, our study suggests that RPL9 KD suppresses B-ALL proliferation and upregulates immunotherapy targets, highlighting the important role of RPL9 as a potential target for conventional and immunotherapy of B-ALL.

## 1 Introduction

B-cell acute lymphocytic leukemia (B-ALL) has made revolutionary progress through T-cell based immunotherapy, e.g., bispecific T-cell conjugates (BiTE) therapy, CD19 chimeric antigen receptor (CAR) T-cell therapy (1, 2). However, multiple clinical trials have shown that most cases of B-ALL, especially those with relapsed and refractory (R/R) B-ALL, do not have a long-term remission rate after CAR-T cell therapy, and new treatment strategies are urgently needed (3–5). In recent years, natural killer (NK) cells, as an important innate immune cells with low toxicity and side effects, have begun to be tested and applied in preclinical studies for the treatment of B-ALL (6). However, B-ALL cells often downregulate natural killer cell family 2 member D ligands (NKG2DLs), e.g., MICA/B, resist and evade the cytotoxicity of NK cells (7, 8). In addition, compared with myeloid leukemia cells, B-ALL cells have a faster proliferation rate (9), and higher ribosome biosynthesis in B-ALL cells likely plays a pivotal role. Therefore, it is necessary to conduct in-depth research on the regulation mechanism of ribosome biosynthesis in B-ALL cells and the specific mechanism of evasion of NK cell-mediated cytotoxicity, which can provide theoretical basis for B-ALL therapy.

Ribosomal protein L9 (RPL9), an important ribosomal structural protein, is a critical component of the 60S ribosomal subunit (10). Recent studies have shown that RPL9 plays a direct regulatory role in ribosome biosynthesis, regulating pre-rRNA processing and cellular energy metabolism. It also has regulatory functions independent of ribosome biosynthesis (10–12). In terms of functions beyond ribosomes, numerous studies have reported that RPL9 is overexpressed and positively regulates tumor progression in several cancers, including colorectal cancer (CRC) (11) and hepatocellular carcinoma (HCC) (12). Specifically, in CRC, Baik et al. found that RPL9 KD leads to the upregulation of several tumor suppressor genes, e.g., KLF6 and ATF3, which contributes to delayed cell cycle progression and enhanced apoptosis (11). In HCC, Li et al. found that silencing RPL9 decreases the expression of several miRNAs, such as miR-24-3p and miR-185-5p, which in turn suppresses the proliferation of HCC cell both *in vivo* and *in vitro (* 12). Regarding ribosome biosynthesis, recent studies have identified mutations in the RPL9 protein in diamond-blackfan anemia (DBA), where aberrant expression of RPL9 primarily impedes pre-rRNA processing, suppresses ribosome biosynthesis, and accelerates DBA progression (10). However, the role of RPL9 in the progression of B-ALL remains unclear.

This study found that the ribosomal structural protein RPL9 was overexpressed in both B-ALL cell lines and patient samples. RPL9 KD significantly suppressed B-ALL cell proliferation both *in vitro* and *in vivo*, while enhancing apoptosis. Mechanistically, RPL9 KD in B-ALL cells induced serious nucleolar stress, inhibited ribosome biogenesis, and activated the p53 signaling pathway. In addition, the study found that FTO, which is an upstream regulator of m^6^A-RPL9, alleviated the activation of p53 signaling pathway in RPL9-KD cells when overexpressed. In addition, our results showed that RPL9 KD further promoted the upregulation of MICA/B on the membrane surface. These results suggest that RPL9 KD may play a key role in inhibiting the progression of B-ALL and potentially enhancing the immune response mediated by NK cells.

## 2 Materials and methods

### 2.1 Human B-ALL cells culture and infection

Human B-ALL cell lines (NALM-6, BALL-1, and RS4;11) were cultured in RPMI-1640 medium with 10% FBS, 2 mM L-glutamine, 100 U/mL penicillin and 100 μg/mL streptomycin. KOPN-8 human B-ALL cells were cultured in IMDM medium with 10% FBS, 2 mM L-glutamine, 100 U/mL penicillin, and 100 μg/mL streptomycin. To achieve RPL9 KD in human B-ALL cells, we incorporated the shRNA sequence targeting RPL9 (GCAATCAGACTGTCGACATTC) into a lentiviral vector and packaged, concentrated, and purified it in 293T cells. ShCtrl lentivirus was utilized as a control. Subsequently, four distinct human B-ALL cell lines (NALM-6, KOPN-8, RS4;11 and B-ALL) were seeded into 96-well plates, and both shRPL9 and shCtrl lentivirus were added into the medium. The culture medium was replaced after 12 hours, and the cells were further cultured for a period ranging from 1 day to 5 day. In certain experiments, overexpression (OE)-FTO lentivirus was also introduced to infect the human B-ALL cells.

### 2.2 Human samples

Bone marrow (BM) samples were collected from B-ALL patients and individuals with nutritional anemia (excluding hematopoietic malignancies) at Xi’an International Medical Center. This was completed with the approval of the Ethics Committee of Xi’an International Medical Center and Northwestern Polytechnical University. CD19^+^ B cells were isolated by human CD19 microbeads (Miltenyi Biotec, USA).

### 2.3 RNA sequencing

RNA sequencing (RNA-seq) was performed according to the previous describe (13, 14). Simplicity, total RNA was extracted from NALM-6 cells (3 × 10^6^/well) utilizing TRIzol reagent. RNA-seq and the data analysis were performed with a commercial service provided by the Gene Denovo Biotechnology Company (Guangzhou, China).

### 2.4 Flow cytometry

The steps of cell surface staining are as follows. Initially, Fc receptor blockade was conducted on the cells for 10 minutes. The cells were then stained in the dark at 4°C for 30 minutes using either PE-conjugated anti-CD19 or APC-conjugated anti-MICA/B antibodies. Following staining, the cells underwent a single wash with PBS, and the percentage of CD19 or/and MICA/B positive cells was quantified utilizing a flow cytometer (Beckman, USA). Then, FlowJo software (FlowJo, LLC, USA) was used to analyze the flow cytometry data.

Intracellular staining utilized the fixation/permeabilization kit (BD Biosciences, USA). Cells were suspended in a fixation/permeabilization solution at 4°C in the dark, washed twice with 1 × BD perm/wash buffer, and then resuspended in BD perm/wash buffer containing antibodies (anti-p21, anti-RPL9, or anti-Myc) at 4°C for 1 hour. After two washes with 1 × BD perm/wash buffer, resuspend in staining buffer with fluorochrome-conjugated secondary antibody and incubate at 4°C for 30 minutes. Protein abundance was evaluated by analyzing mean fluorescence intensity (MFI) using Flow cytometry and FlowJo software.

### 2.5 *In vivo* B-ALL cell transplantation

In the NCG mouse xenotransplantation model, RS4;11 cells or patient B-ALL cells were infected with shCtrl lentivirus or shRPL9 lentivirus. Then, 1 × 10^6^ live cells were injected into NCG mice through the tail vein (day 0), and the survival rate of the mice was recorded. In the RS4;11 tumor model, human CD19^+^ RS4;11 cells from the bone marrow, spleen, liver, and lung were analyzed by flow cytometry in week 7.

### 2.6 Other materials and methods are shown in supplementary materials

### 2.7 Statistical analysis

Statistical analyses were performed using GraphPad Prism 7.0 (USA). To compare two groups, the unpaired t-test was applied. Survival functions were estimated using the Kaplan-Meier method. Results are presented as mean ± SD, with statistical significance set at P < 0.05.

## 3 Results

### 3.1 RPL9 shows elevated expression levels in both B-ALL cell lines and patient samples

**Figure 1:**
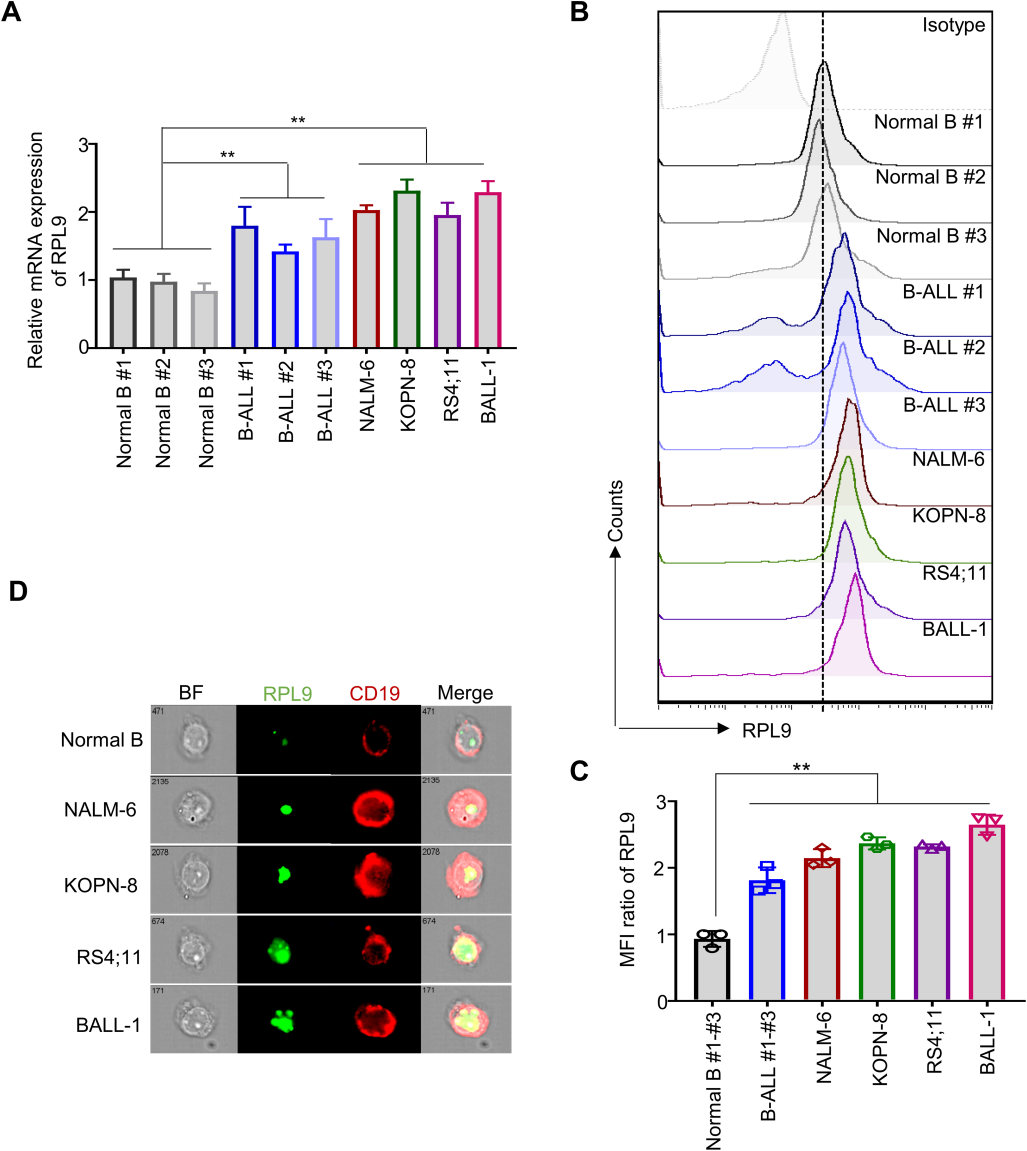
RPL9 shows elevated expression levels in both B-ALL cell lines and patient samples. **(A)** The mRNA expression of human *RPL9* in normal B cells, B-ALL patient B cells, and B-ALL cell lines (NALM-6, KOPN-8, RS4;11 and B-ALL cells). **(B)** The protein expression of human RPL9 in normal B cells, B-ALL patient B cells, and B-ALL cell lines (NALM-6, KOPN-8, RS4;11 and BALL-1) was detected by flow cytometry. **(C)** The relative mean fluorescence intensity (MFI) of **(B)**. **(D)** The protein expression of human RPL9 in normal B cells and B-ALL cell lines (NALM-6, KOPN-8, RS4;11 and B-ALL cells) was detected by using imaging flow cytometry. Bars represent means ± SD, “**” P<0.01.

Recent studies have clarified the role of RPL9 mutations or abnormal expressions in the progression of diseases, like DBA (10), HCC (12), and CRC (11). To investigate the function of RPL9 in B-ALL, we first assessed the mRNA expression levels of RPL9 in samples from B-ALL patients, B-ALL cell lines, and normal B cells. Our analysis showed that RPL9 mRNA expression was significantly higher in three primary B-ALL cells and four B-ALL cell lines (NALM-6, KOPN-8, RS4;11 and BALL-1) compared to normal B cells (**Figure 1A**). Flow cytometry analysis revealed significantly higher RPL9 protein expression in B-ALL patients and cell lines compared to normal B cells (**Figures 1B, C**). Imaging flow cytometry further validated that RPL9 expression was significantly elevated in B-ALL cell lines (NALM-6, KOPN-8, BALL-1, and RS4;11) compared to normal B cells (**Figure 1D**). In summary, our findings indicate a comprehensive upregulation of RPL9 in both B-ALL cell lines and patient samples.

### 3.2 RPL9 knockdown impedes the proliferation of human B-ALL cells

**Figure 2:**
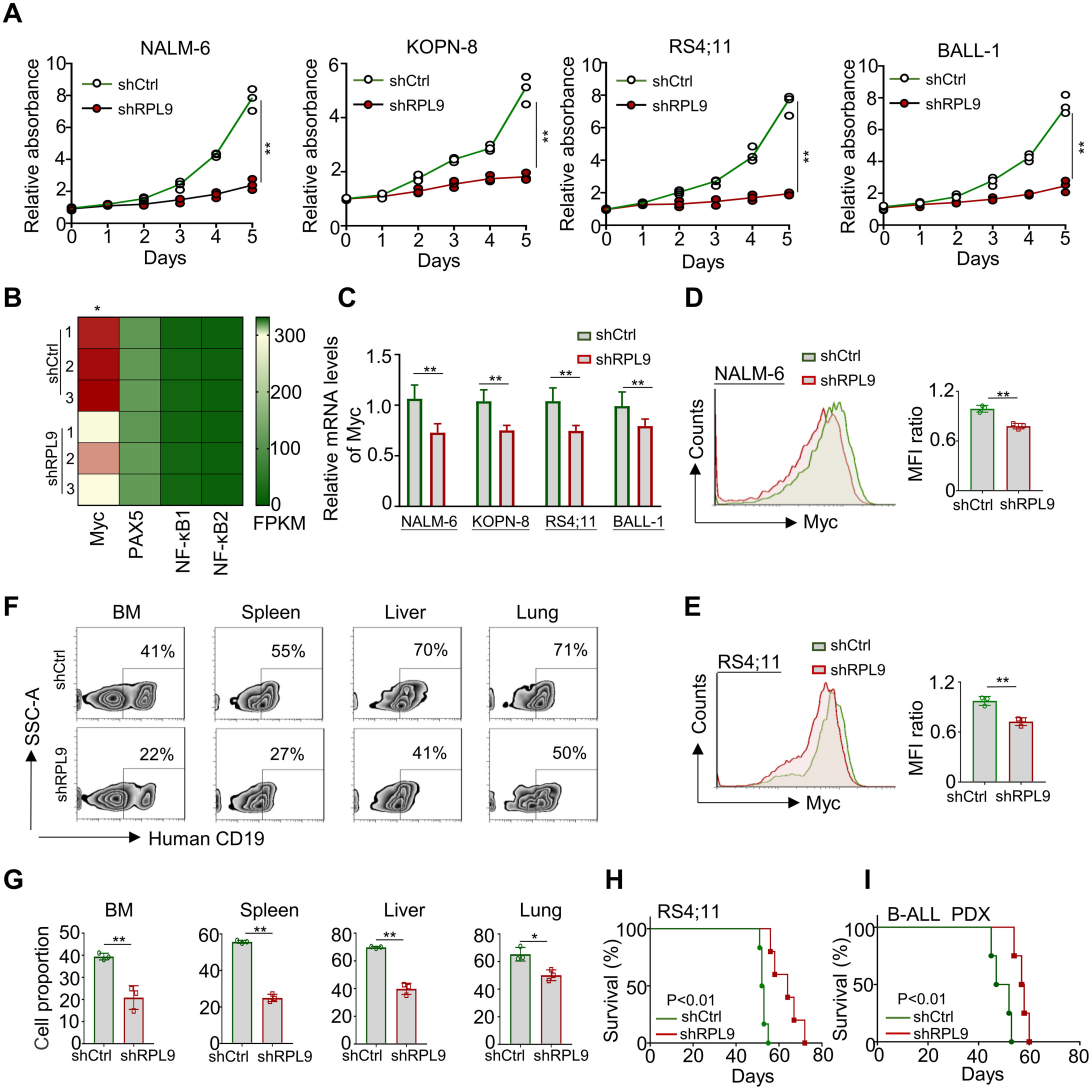
RPL9 knockdown inhibits the proliferation of B-ALL cells. **(A)** NALM-6, KOPN-8, RS4;11 and B-ALL cells were infected with shRPL9 lentivirus or their control lentivirus, and cultured for 5 days. Cell proliferation was detected by CCK8 method. **(B)** The FPKM of oncogenic transcription factor in shRPL9 NALM-6 cells and shCtrl NALM-6 cells. **(C)** NALM-6, KOPN-8, RS4;11 and B-ALL cells were infected with shRPL9 lentivirus or their control lentivirus, after 72 hours, the mRNA expression of Myc was detected through RT-qPCR. **(D, E)** NALM-6, and RS4;11 cells were infected with shRPL9 lentivirus or their control lentivirus, after 72 hours, the protein expression of Myc were detected through flow cytometry. **(F, G)** single cell suspensions of BM, spleen, liver and lung from B-ALL mouse model were analyzed by flow cytometry **(F)**, and the percentage of human CD19^+^ B cells were quantitatively compared **(G)**. **(H, I)** Survival of NCG mice which injected with shRPL9 RS4;11 cells or shRPL9 patient-derived B-ALL cells and their control cells. N=3-6, Bars represent means ± SD, “**” P<0.01, “*” P<0.05.

To elucidate the specific regulatory role of RPL9 in B-ALL cells, we conducted experiments wherein B-ALL cells were transduced with control lentivirus (shCtrl) or RPL9-targeting lentivirus (shRPL9). Firstly, we assessed the KD efficiency of RPL9 by using RT-qPCR and flow cytometry. The findings indicated a significant reduction to approximately 20-30% in both mRNA and protein levels of RPL9 in the shRPL9 group compared to the shCtrl group, confirming effective RPL9 knockdown (**Supplementary Figures 1A, B**). Subsequently, we examined the regulation role of RPL9 in B-ALL cell proliferation. The results showed that a marked decrease in the proportion of proliferating cells in shRPL9 group relative to the shCtrl group, particularly in days 3, 4 and 5 (**Figure 2A**). The mRNA and protein expression levels of the oncogenic transcription factor Myc, a crucial regulator of B-ALL proliferation, were significantly reduced supporting the aforementioned results (**Figures 2B–E**). These findings indicate that RPL9 enhances B-ALL proliferation *in vitro*.

To assess the impact of RPL9 KD on B-ALL proliferation *in vivo*, RS4;11 cells and primary B-ALL cells from a patient were infected with shCtrl lentivirus or shRPL9 lentivirus. Subsequently, the infected cells were transplanted into NCG immunodeficient mice. In a portion of RS4;11 tumor model, at week 7, flow cytometry was used to quantify CD19^+^B-ALL cells in BM, spleen, liver and lung. The survival rates of tumor-bearing mice were assessed in both the RS4;11 tumor model and the primary B-ALL tumor model. The findings indicated that RPL9 KD markedly decreased cell proliferation in the BM, spleen, liver, and lung compared to the control group (**Figures 2F, G**). In addition, compared with the control mice, the survival time of RPL9 KD mice was significantly prolonged (**Figures 2H, I**). The results demonstrate that RPL9 KD markedly suppressed B-ALL cell proliferation *in vivo*.

### 3.3 RPL9 knockdown promote the apoptosis of human B-ALL cells

**Figure 3:**
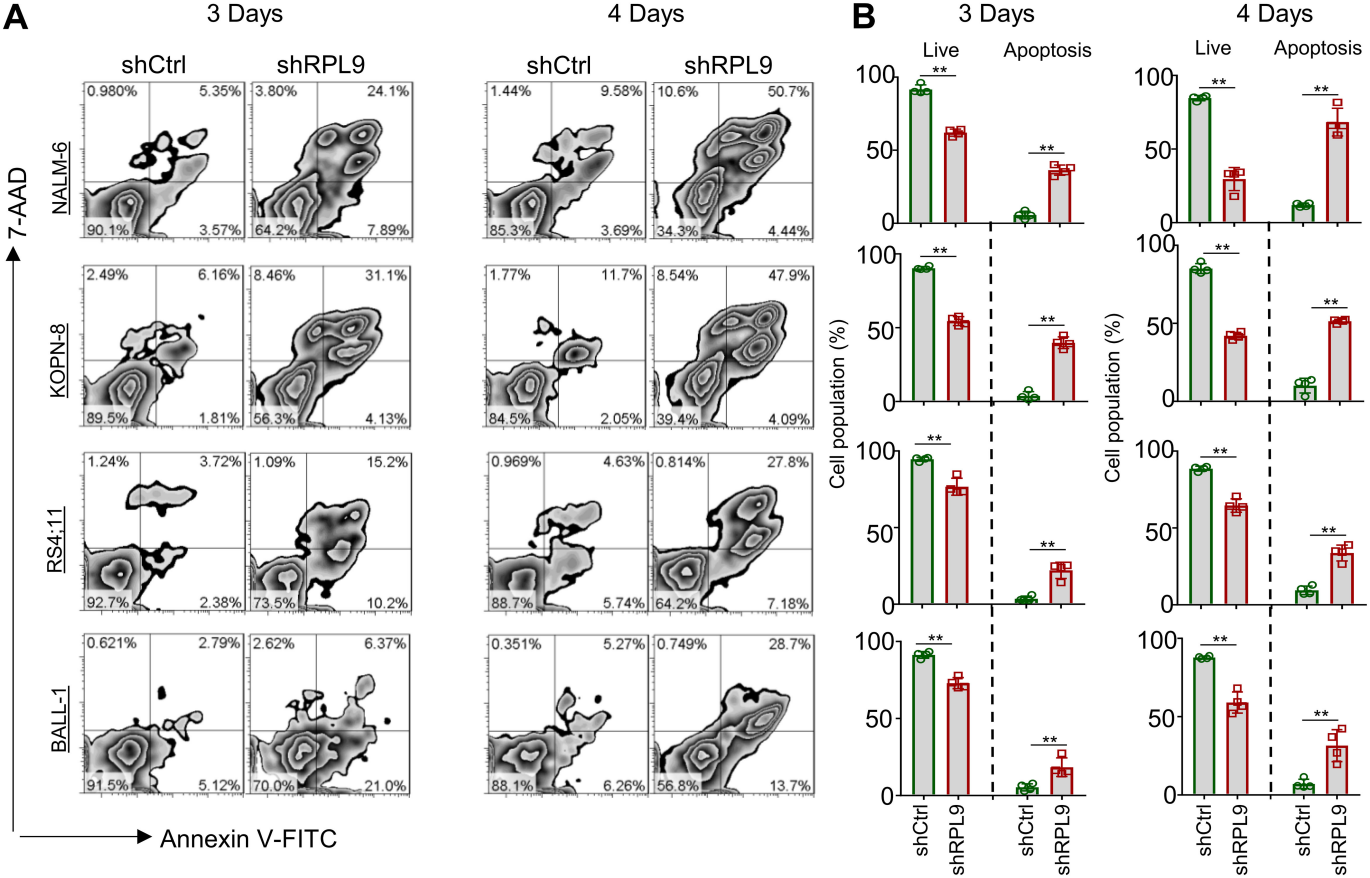
RPL9 knockdown promote the apoptosis of B-ALL cells. **(A, B)** NALM-6, KOPN-8, RS4;11 and BALL-1 cells were infected with shCtrl or shRPL9 lentivirus, after 3 day and 4 days, cell apoptosis were analyzed by flow cytometry. N=4, Data represent mean ± SD, “**” P < 0.01.

We analyzed the effect of RPL9 KD on B-ALL cell apoptosis using flow cytometry, revealing a significant decrease in the proportion of live (Annexin-V^-^/7-AAD^-^) cells in the shRPL9 group compared to the shCtrl group. On day 3, there was a significant rise in the percentages of early apoptotic (Annexin-V^+^/7-AAD^-^) and late apoptotic (Annexin-V^+^/7-AAD^+^) cells (**Figures 3A, B**). Furthermore, this apoptotic trend persisted on the day 4, with the effects of RPL9 KD being more pronounced than those observed on the day 3 (**Figures 3A, B**). These findings collectively demonstrate that RPL9 KD substantially promotes apoptosis in B-ALL cells.

### 3.4 Knockdown of RPL9 activates the p53 signaling pathway

**Figure 4:**
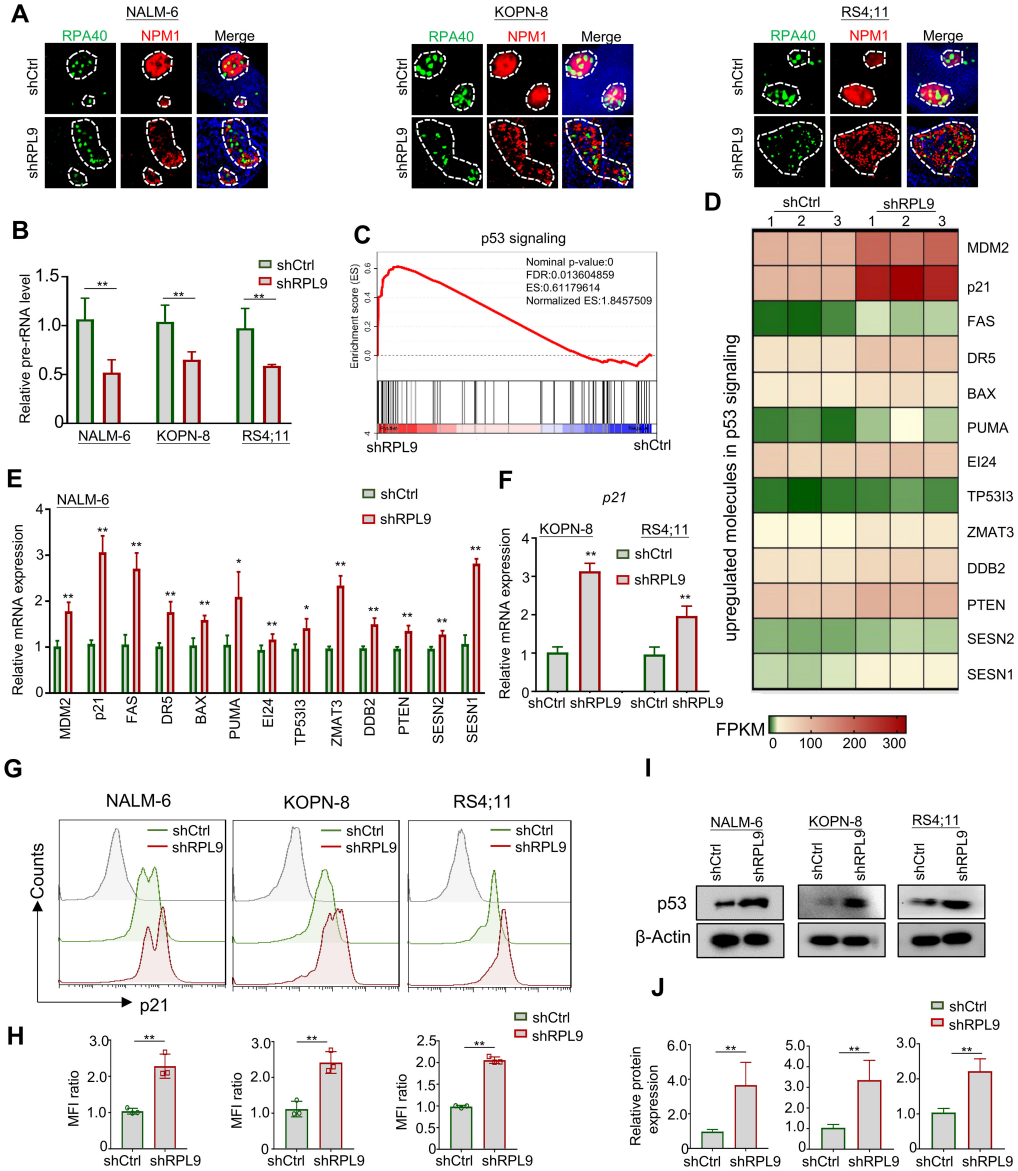
RPL9 knockdown activate the p53 signaling pathway. **(A)** NALM-6, KOPN-8 and RS4;11 cells were infected with shCtrl or shRPL9 lentivirus, and subjected to immunofluorescence staining of nuclear markers RPA40 and NPM1 (single cell nucleus). **(B)** NALM-6, KOPN-8 and RS4;11 cells were infected with shCtrl or shRPL9 lentivirus, and the expression of pre-rRNA were detected. **(C)** GSEA analysis of p53 signaling in shRPL9 vs shCtrl group. **(D)** Signature upregulated genes of p53 signaling between shRPL9 and shCtrl group. **(E)** NALM-6 cells were infected with shCtrl or shRPL9 lentivirus, after 72 hours, the mRNA expression of 13 molecules in p53 signaling were shown. **(F)** KOPN-8 and RS4;11 cells were infected with shCtrl or shRPL9 lentivirus, after 72 hours, the mRNA expression of p21 were shown by using RT-qPCR. **(G, H)** NALM-6, KOPN-8 and RS4;11 cells were infected with shCtrl or shRPL9 lentivirus, after 72 hours, the protein expression of p21 were shown through flow cytometry. **(I, J)** NALM-6, KOPN-8 and RS4;11 cells were infected with shCtrl or shRPL9 lentivirus, after 72 hours, the protein expression of p53 were shown through immunoblot. N=3, Data represent mean ± SD, “**” P < 0.01, “*” P<0.05.

To elucidate the molecular mechanism of RPL9 KD in inhibiting B-ALL cell proliferation and inducing apoptosis, firstly, we evaluated the nucleolar stress state through immunofluorescence staining of nucleolar localization proteins RPA40 and NPM1. Our findings demonstrate that RPL9 KD altered the normal elliptical shape, leading to fragmented morphology in three B-ALL cell lines compared to the control group (**Figure 4A**). The above results indicate that the knockdown of ribosomal subunit structural protein RPL9 can induce nucleolar stress. Our further analysis revealed a marked decrease in pre-rRNA expression in shRPL9 B-ALL cells compared to shCtrl cells (**Figure 4B**). Previous studies indicated that nucleolar stress-induced displacement of NPM1 protein activates the p53 signaling pathway, which is otherwise suppressed by MDM2 (15). KEGG and GSEA of our RNA-seq data revealed that RPL9 KD significantly activates the p53 signaling pathway, particularly enhancing the expression of 13 positive regulators within this pathway, as indicated by increased FPKM levels in the RPL9 KD group (**Supplementary Figure 2**, **Figures 4C, D**). RT-qPCR further confirmed that RPL9 KD significantly upregulated the mRNA expression of these 13 molecules of p53 signaling in NALM-6 cells (**Figure 4E**). In KOPN-8 and RS4;11 cell lines, the expression of p21 mRNA was also significantly increased (**Figure 4F**).

Subsequently, we assessed the impact of RPL9 KD on the protein expressions of p21 and p53 using flow cytometry and immunoblot analysis. The results revealed that, relative to the control group, RPL9 KD markedly promote the protein expression of p21 (**Figures 4G, H**). RPL9 KD notably elevated p53 protein expression relative to the control group (**Figures 4I, J**). The findings indicate that RPL9 KD trigger nucleolar stress and activates the p53 signaling pathway in B-ALL cells.

### 3.5 RPL9 knockdown mediated p53 signaling activating can be inhibited by FTO overexpression

Our previous research demonstrated that *RPL9* have m^6^A modification, and that the removal of the m^6^A modification through FTO overexpression significantly increase both mRNA and protein levels of RPL9. To further explore whether FTO overexpression can suppress the activation of p53 signaling induced by RPL9 KD, we overexpress FTO in RPL9 KD B-ALL cells, and subsequently assessed the mRNA and protein expression of p21 and p53. Immunoblot analysis demonstrated the overexpression efficiency of OE-FTO (**Supplementary Figure 3A**). RT-qPCR analysis demonstrated that the OE-FTO + shRPL9 group significantly decreased p21 mRNA expression compared to the shRPL9 group (**Supplementary Figure 3B**). Flow cytometry and immunoblot analyses demonstrated that the OE-FTO + shRPL9 group significantly suppressed the protein expression of both p21 and p53 compared to the shRPL9 group (**Supplementary Figures 3C, D**). In conclusion, these findings confirm that the activation of p53 signaling mediated by RPL9 KD can indeed be suppressed by the overexpression of FTO.

### 3.6 Knocking down RPL9 increase MICA/B expression in B-ALL cells, potentially enhancing their sensitivity to NK cell-mediated cytotoxicity

**Figure 5:**
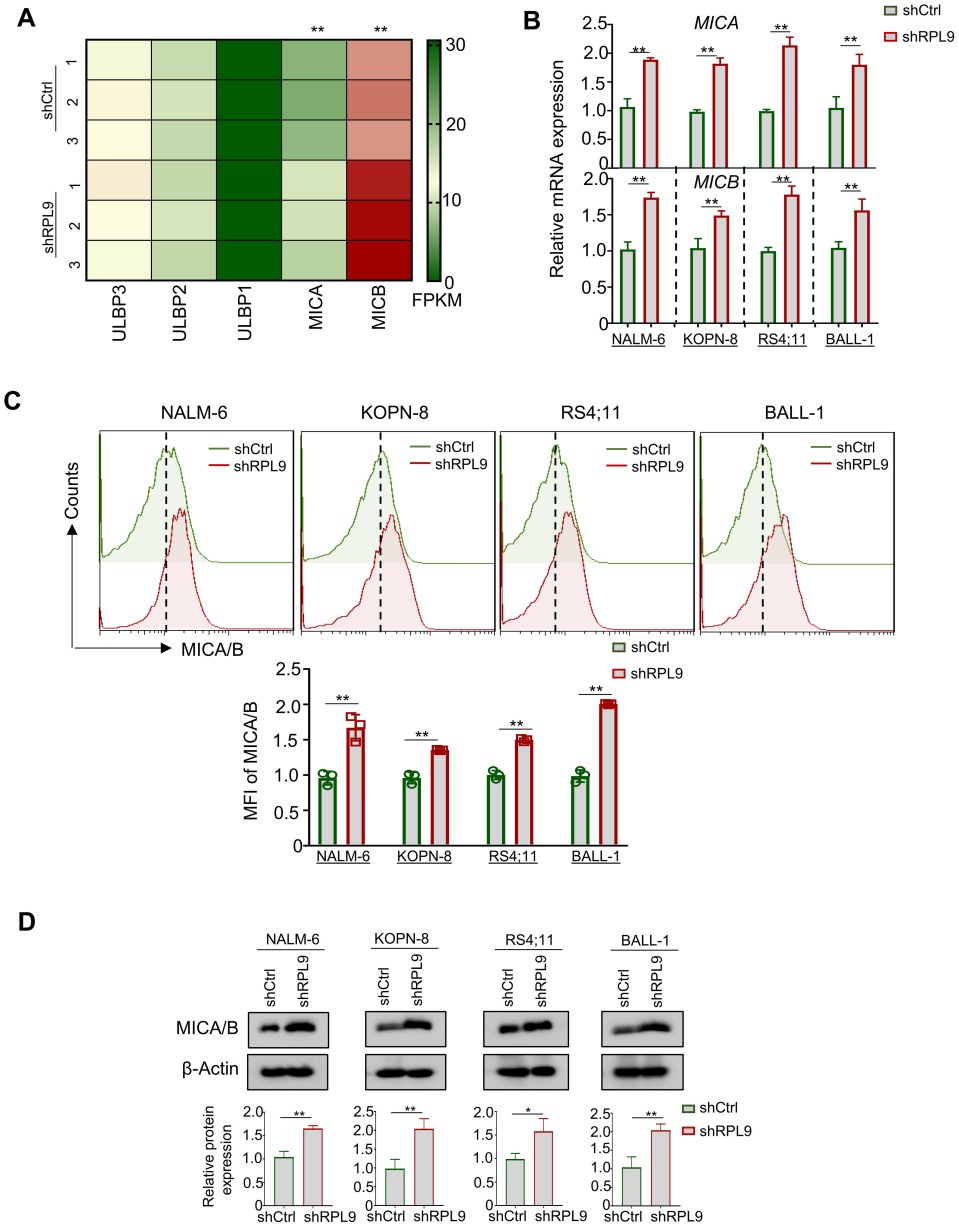
RPL9 knockdown promote the expression of MICA/B. **(A)** NALM-6 cells were infected with shRPL9 lentivirus or its control lentivirus for 72 hours, and the FPKM of NKG2DLs mainly ULBP1, ULBP2, ULBP3, MICA and MICB, were determined by RNA-seq. **(B)** NALM-6, KOPN-8, RS4;11 and BALL-1 cells were infected with shRPL9 lentivirus or its control, and MICA and MICB mRNA expressions were determined by RT-qPCR. **(C)** NALM-6, KOPN-8, RS4;11 and BALL-1 cells were infected with shRPL9 lentivirus or its control, the proportion of MICA/B positive cells were determined by flow cytometry. **(D)** NALM-6, KOPN-8, RS4;11 and BALL-1 cells were infected with shCtrl lentivirus or shRPL9, after 72 hours, the protein expression of MICA/B were detected using immunoblot. Bars represent means ± SD, N=3, “**” P < 0.01, “*” P < 0.05.

Research indicates that activating p53 signaling in tumor cells increases NKG2DLs, thereby boosting NK cell-mediated cytotoxicity (16). Anti-tumor immune cells (e.g., NK cells), can recognize NKG2DLs on tumor cells, primarily ULBP1, ULBP2, ULBP3, MICA, and MICB (17). In this study, RNA-seq analysis revealed RPL9 KD in NALM-6 cells significantly upregulated MICA and MICB mRNA expressions compared to control NALM-6 cells, indicating a potential enhancement of NK cell-mediated anti-tumor immune responses in B-ALL cells (**Figure 5A**). Four B-ALL cell lines were infected with shRPL9 and shCtrl lentivirus for 72 hours to assess MICA and MICB mRNA and protein expression via RT-qPCR, flow cytometry and immunoblot. The findings indicated a significant increase in mRNA and protein expression of MICA and MICB in the RPL9 KD group compared to the control group (**Figures 5B–D** **).** The findings indicate that RPL9 KD may enhance the sensitivity of B-ALL cells to NK cell-mediated cytotoxicity.

## 4 Discussion

**Figure 6:**
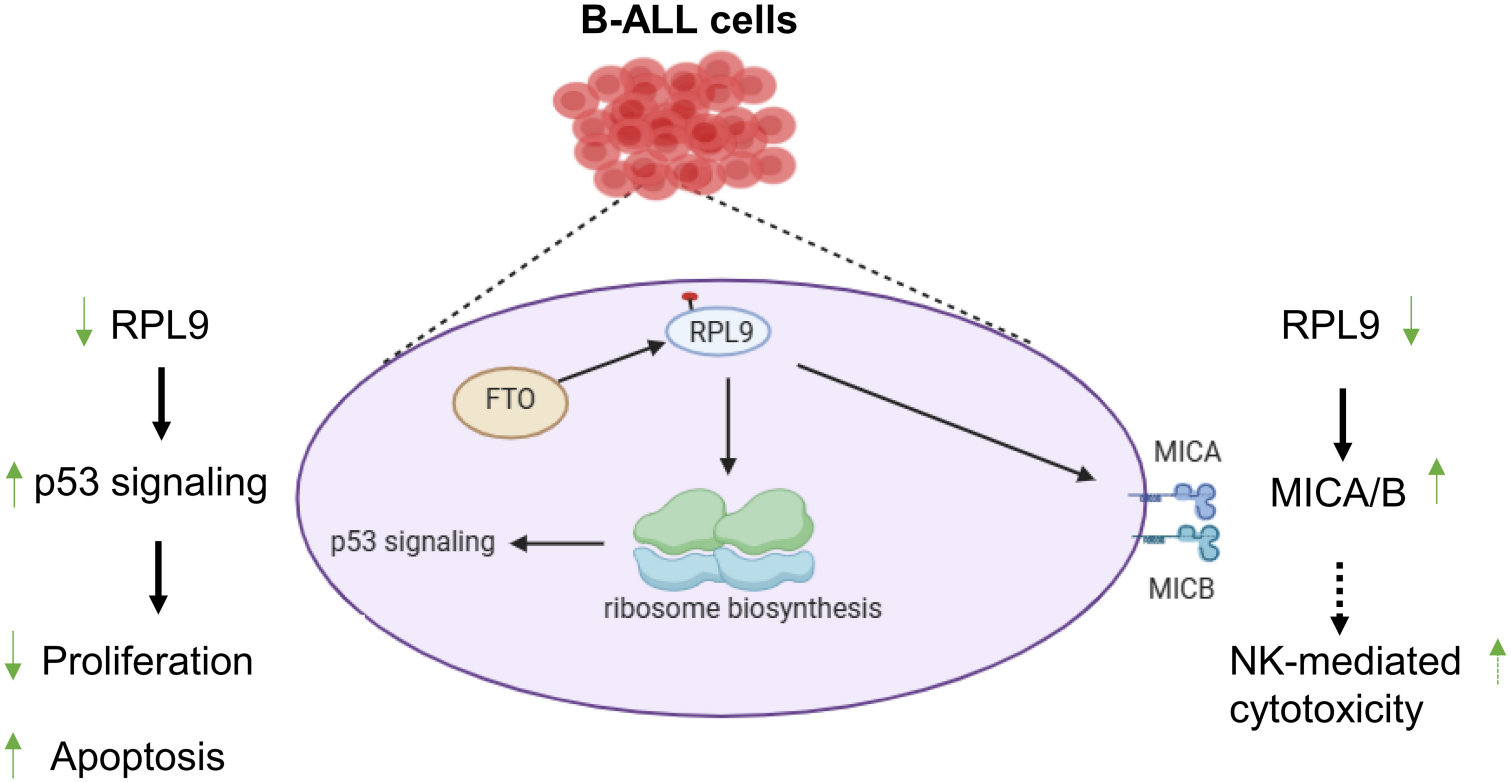
RPL9 is a potential therapeutic target for B-ALL by activating the p53 signaling pathway.

In this study, we reported that RPL9 KD inhibits the proliferation and promote the apoptosis of human B-ALL cells. Mechanistically, we have identified that p53 signaling pathway is an important pathway for RPL9-mediated B-ALL progression. And, overexpression of FTO, which is the key upstream regulator of m^6^A-RPL9, can inhibit the activation of p53 signaling which caused by RPL9 KD. Finally, we also found that RPL9 KD promotes the expression of MICA/B, which are critical activating ligands of NKG2D on NK cells and has been reported as a key downstream molecule of p53 signaling (18). Our observation provides for the first time a new regulatory mechanism and targeted inhibition strategy for RPL9-mediated B-ALL progression, and suggests that targeted inhibition of RPL9 may provide advantages for B-ALL therapy, while also promoting the therapeutic sensitivity of B-ALL to NK cell immunotherapy (**Figure 6**).

Previous researches has demonstrated an association between RPL9 and the progression of malignant disease (10–12). In solid tumors, e.g., HCC and CRC, RPL9 KD has been shown to inhibit cell proliferation and cell cycle progression, while inducing apoptosis in malignant cells (10–12). This effect is mediated through the targeting of various genes, mainly KLF6, ATF3, miR-24-3p, or miR-185-5p (11, 12). These findings suggest that RPL9 functions as a positive regulator in tumor progression. Recently, it has been discovered that RPL9, as an important component of ribosomal subunits, regulates the biosynthesis of ribosomes (10). Recently, our laboratory has found that RPL9 has m^6^A modification and it is one of the key target for FTO-mediated ribosome biosynthesis. FTO KD can increase the m^6^A modification level of RPL9 and promote YTHDF2-mediated degradation of RPL9 mRNA. Concurrently, Myc is recognized as a pivotal oncogenic transcription factor in B-ALL, with its overexpression facilitating the advancement of the disease (19). In this study, we elucidated the specific oncogenic function of RPL9 in B-ALL, demonstrating its role in modulating cellular proliferation and apoptosis phenotypically, as well as influencing the expression of the oncogenic transcription factor Myc at the molecular level. Our findings suggest that RPL9 actively contributes to the progression of B-ALL, and that targeted inhibition of RPL9 in B-ALL cells may represent a promising therapeutic strategy for the treatment of this malignancy.

The infinite proliferation of tumor cells necessitates extensive ribosome biosynthesis to maintain the protein synthesis required for large-scale cellular proliferation (20). Improving ribosome biosynthesis capabilities can facilitate the proliferation of B-ALL cells (21). Conversely, nucleolar stress resulting from ribosome assembly dysfunction induces cell cycle arrest and apoptosis through activation of the p53 signaling. RPL9 serves as a critical structural component of ribosomes (10) and the p53 signaling plays a crucial role in mediating ribosome biosynthesis (22). In 2017, Wlodarski et al. demonstrated that mutations in RPL9 significantly impair ribosome biosynthesis in hereditary bone marrow failure, leading to the dissociation of MDM2 from p53, thereby activating the p53 signaling (23). This finding underscores the pivotal role of p53 signaling in RPL9-mediated ribosome biosynthesis. In our study, we present evidence that knockdown of RPL9 results in dysregulation of ribosome assembly, suppression of ribosome biosynthesis, induction of nucleolar stress, activation of p53 signaling, and consequently, inhibition of B-ALL cell proliferation and promotion of apoptosis.

In the development of hematological malignancies and solid tumors, tumor cells within the tumor microenvironment (TME) can modulate the expression of MHC-I molecules (such as HLA-A/B/C, HLA-E), NKG2D ligands (such as ULBP1/2/3, MICA/B), or PD-L1 through altering intracellular metabolism and ribosome biosynthesis-mediated protein synthesis, enabling them to evade the cytotoxic effects of immune cells, like NK cells (24). Specifically, MICA/B, as an important activating ligand for NKG2D, is typically expressed at low levels on cell membrane surface in hematological malignancies, allowing it to escape NK cell-mediated cytotoxicity (7, 8). Prior studies has shown that reducing ribosomal proteins (RPs) levels can facilitate the expression of MICA/B in tumor cells (25). Consistent with these findings, our study indicates that RPL9 KD enhances MICA/B expression in B-ALL cells, potentially heightening their vulnerability to NK cell-mediated cytotoxicity. Additionally, existing literature indicates that the activation of p53 signaling results in the upregulation of MICA/B expression (18, 26). Our findings confirm that RPL9 knockdown activates p53 signaling, resulting in increased MICA/B mRNA and protein expression on B-ALL cell membranes. Nonetheless, further investigation is required to ascertain whether the RPL9 KD-induced activation of p53 signaling directly influences MICA/B expression in B-ALL cells. While MICA/B may serve as a direct downstream target of p53 signaling, it is also plausible that RPL9 downregulation modulates MICA/B expression through alternative pathways.

In summary, the research we report here reveals the role of RPL9 as a positive regulator of human B-ALL cell proliferation and anti-apoptosis. Our research findings provide a new strategy for how RPL9 KD reduces ribosome biosynthesis by activating p53 signaling, and further activates nucleolar stress in B-ALL cells to prevent proliferation and promote the apoptosis of B-ALL cells. RPL9 KD can also promote the expression of MICA/B, potentially enhancing the sensitivity of B-ALL cells to NK cell-mediated cytotoxicity, which may provide theoretical support for the NK cells immunotherapy on B-ALL.

## Data Availability

The datasets presented in this study can be found in online repositories. The names of the repository/repositories and accession number(s) can be found in PRJNA1226775.
