# Supplementary material for: Ribosomal protein L9 is a potential therapeutic target for B-ALL through the activation of the p53 signaling pathway
Source: Front Immunol. 2025 Mar 27;16:1560706. doi: 10.3389/fimmu.2025.1560706 (PMC11983633; doi:10.3389/fimmu.2025.1560706)
Supplement: Supplementary file 1 [file DataSheet1.docx]

**Ribosomal protein L9 is a potential therapeutic target for B-ALL through the activation of the p53 signaling pathway**

Xinxin Li ^a,b,*^, Wenting Meng^c^, Xi Wang^c^, Siyong Huang^d^, Jianbin Wang^e^, Han Liang^f^, Dailing Si^f^

^a^Xi’an Key Laboratory of Stem Cell and Regenerative Medicine, Institute of Medical Research, Northwestern Polytechnical University, Xi’an 710072, Shaanxi, China;

^b^Research & Development Institute of Northwestern Polytechnical University in Shenzhen, Shenzhen Guangdong, 518000, China;

^c^Department of Neurosurgery, Xijing Hospital, Fourth Military Medical University, Xi’an 710032, Shaanxi, China;

^d^Department of Hematology, Xi’an International Medical Center Hospital, Xi’an 710100, Shaanxi, China;

^e^Department of Medical Genetics and Developmental Biology, Fourth Military Medical University, Xi’an 710032, Shaanxi, China;

^f^Analysis & Testing Laboratory for Life Sciences and Medicine, Fourth Military Medical University, Xi’an 710032, Shaanxi, China.

^*^For correspondence, please contact Xinxin Li at the Institute of Medical Research, Northwestern Polytechnical University, Youyi West Road #127, Xi’an 710072, China, Email: lixinxin1989123@nwpu.edu.cn.

**MATERIALS AND METHODS**

**B-ALL cell proliferation and apoptosis**

Cell proliferation was assessed using the CCK-8 kit (Beyotime, China). In particular, 10 μl of CCK-8 reagent was added to cells in 96-well plates that were infected with shRPL9 lentivirus or shCtrl lentivirus. Following a 2 hours incubation at 37 °C, absorbance at 450 nm was measured.

Cell apoptosis was assessed with FITC-Annexin-V and 7-AAD (BD Bioscience, USA). B-ALL cells were washed twice with cold PBS and resuspended in 1× binding buffer. The solution (100 μl) was transferred to a 1.5 ml culture tube and mixed gently with 5 µl FITC-Annexin-V and 2 µl 7-AAD. The cells were kept in the dark at room temperature (25°C) for 15 minutes. Subsequently, 400 μl of 1 ×binding buffer was added to each tube. The percentage of live cells and total (early and late) apoptotic cells were analyzed using flow cytometry within 1 hour.

**Reverse transcription-quantitative polymerase chain reaction (RT-qPCR)**

Total RNA from B-ALL cells were extracting using TRIzol reagent, followed by cDNA synthesis with a reverse transcription kit. RT-qPCR was conducted using a SYBR Premix Ex Taq^TM^ II Kit on a ABI Quantstudio system (Thermo Fisher Scientific, USA) . Refer to Supplementary Table 1 for primer sequences.

**Immunofluorescence staining**

Incubate the climbing slice in a 24-well plate with poly-D-lysine for 6 hours. Wash with ddH2O, inoculate cells onto the slide, and culture for 12 hours for cell adhesion. Remove the medium, wash with PBS, fix with paraformaldehyde for 30 minutes, and rinse with PBS. Apply PBS with 5% BSA and 0.3% TritonX-100 for 10 minutes, followed by a 2 hours blocking step. Incubate overnight at 4°C with primary antibody (anti-NPM1 or anti-RPA40). Rinse with PBS, incubate with fluorescent secondary antibody in the dark for 2 hours, washed again with PBS, and apply DAPI for 15 minutes. Subsequently, cells were rinsed with PBS and sealed using 75% glycerol. Finally, cells were observed using dual super-resolution confocal system (ZEISS Elyra 7, Germany).

**Immunoblotting**

Equal numbers of cells were washed once with PBS and lysed using either 1×SDS buffer or RIPA lysis buffer with PMSF. Equal amounts of protein lysates from each sample were resolved on SDS-PAGE gels, followed by transfer onto polyvinylidene fluoride (PVDF) membranes. Immunoblotting was conducted using an enhanced chemiluminescence (ECL) substrate (Thermo Fisher Scientific, USA), and images were acquired with the chemiluminescence imaging system. The protein abundance of target proteins was normalized to β-actin and further adjusted relative to the control group using ImageJ software for protein quantification analysis.

**Supplementary Table 1**

| **Supplementary Table 1. PCR primers used in the study** | |
| --- | --- |
| **Name/Target gene** | **Primer sequence** |
| Human RPL9 | F: 5’-GACGCACAGTTATCGTGAAGGG-3’; |
|  | R: 5’-CAAATAGTCCGAACGGTAGCCAG-3’ |
| Human Myc | F: 5’-CCTGGTGCTCCATGAGGAGAC-3’; |
|  | R: 5’-CAGACTCTGACCTTTTGCCAGG-3’ |
| Human MDM2 | F: 5’-TGTTTGGCGTGCCAAGCTTCTC-3’; |
|  | R: 5’- TGTTTGGCGTGCCAAGCTTCTC-3’ |
| Human p21 | F: 5’- AGGTGGACCTGGAGACTCTCAG -3’; |
|  | R: 5’- TCCTCTTGGAGAAGATCAGCCG -3’ |
| Human FAS | F: 5’-GGACCCAGAATACCAAGTGCAG-3’; |
|  | R: 5’-GTTGCTGGTGAGTGTGCATTCC-3’ |
| Human DR5 | F: 5’-AGCACTCACTGGAATGACCTCC-3’; |
|  | R: 5’-GTGCCTTCTTCGCACTGACACA-3’ |
| Human BAX | F: 5’-TCAGGATGCGTCCACCAAGAAG-3’; |
|  | R: 5’-TGTGTCCACGGCGGCAATCATC-3’ |
| Human PUMA | F: 5’-ACGACCTCAACGCACAGTACGA-3’; |
|  | R: 5’-CCTAATTGGGCTCCATCTCGGG-3’ |
| Human EI24 | F: 5’-GCAAGTAGTGTCTTGGCACAGAG-3’; |
|  | R: 5’-CAGAACACTCCACCATTCCAAGC-3’ |
| Human TP53I3 | F: 5’-CACCAGTTTGCTGAGGTCTAGG-3’; |
|  | R: 5’-CCTGGATTTCGGTCACTGGGTA-3’ |
| Human ZMAT3 | F: 5’-GCTCTGTGATGCCTCCTTCAGT-3’; |
|  | R: 5’-TTGACCCAGCTCTGAGGATTCC-3’ |
| Human DDB2 | F: 5’-CCAGTTTTACGCCTCCTCAATGG-3’; |
|  | R: 5’-GGCTACTAGCAGACACATCCAG-3’ |
| Human PTEN | F: 5’-TGAGTTCCCTCAGCCGTTACCT-3’; |
|  | R: 5’-GAGGTTTCCTCTGGTCCTGGTA-3’ |
| Human SESN2 | F: 5’-AGATGGAGAGCCGCTTTGAGCT-3’; |
|  | R: 5’-CCGAGTGAAGTCCTCATATCCG-3’ |
| Human SESN1 | F: 5’-TCACAGTGTGGATGAGATGCCG-3’; |
|  | R: 5’-CTCGACATTCCTGTAACTGCCTC-3’ |
| Human pre-rRNA | F: 5’- GCCTTCTCTAGCGATCTGAGAG-3’; |
|  | R: 5’- CCATAACGGAGGCAGAGACA-3’ |
| Human MICA | F: 5’-CCACCAGGATTTGCCAAGGAGA-3’; |
|  | R: 5’-CTGCCAATGACTCTGAAGCACC-3’ |
| Human MICB | F: 5’-GGAATGGAACCTACCAGACCTG-3’; |
|  | R: 5’-CTGTCCGTTGACTCTGAAGCAC-3’ |


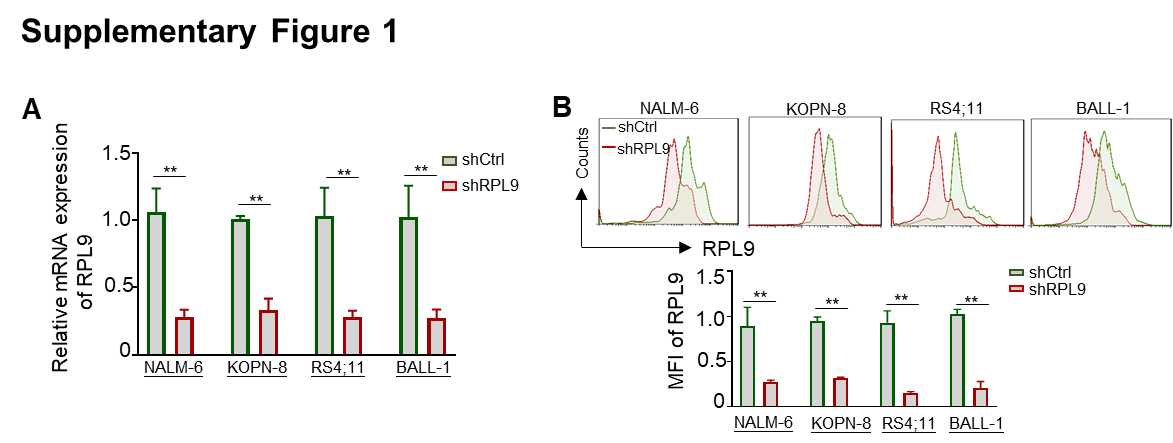


**Supplementary Figure 1. (A-B)** NALM-6, KOPN-8, RS4;11 and B-ALL cells were infected with shRPL9 lentivirus or their control lentivirus. The knockdown efficacy were detected through RT-qPCR and flow cytometry by using CoraLite® Plus 488-conjugated RPL9.


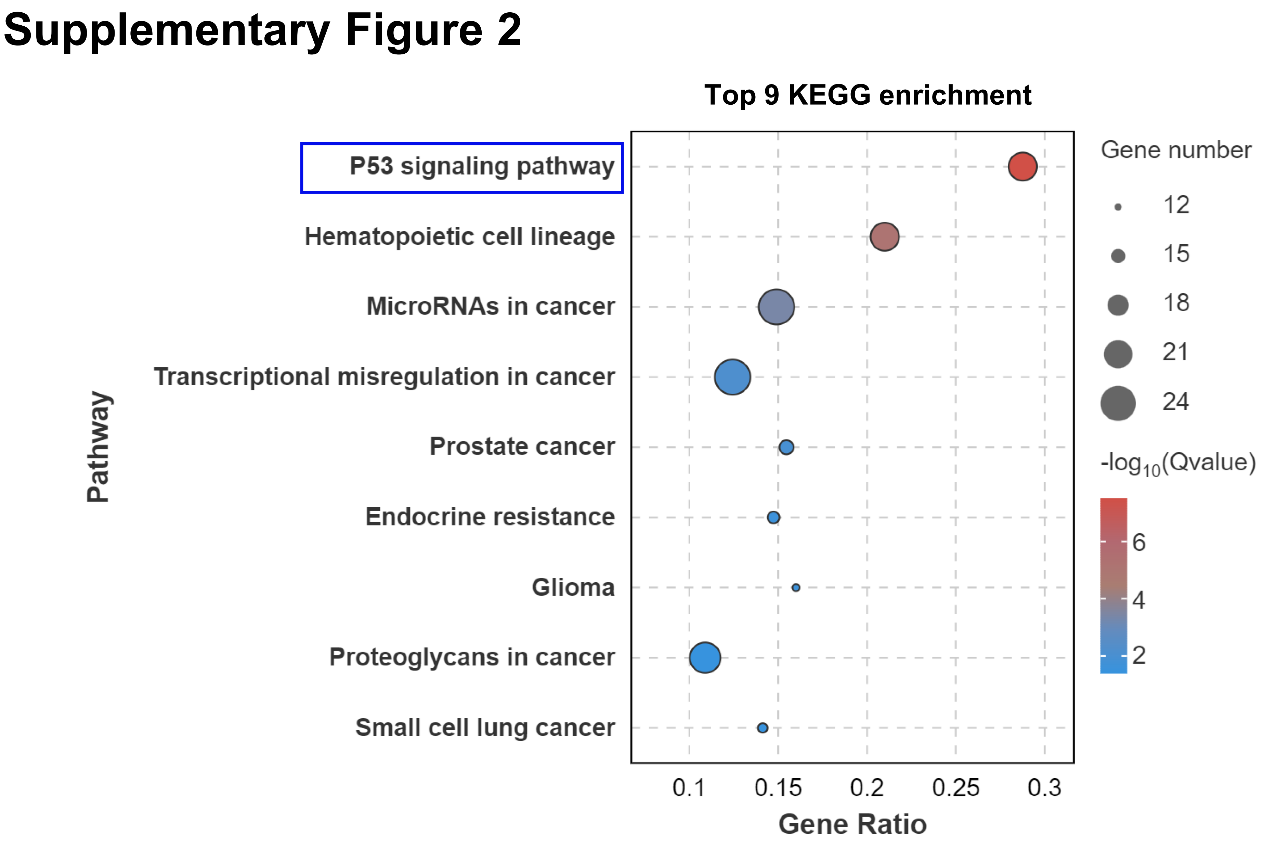


**Supplementary Figure 2.** The KEGG analysis of top 9 significantly changed pathway in shRPL9 vs shCtrl group.

**
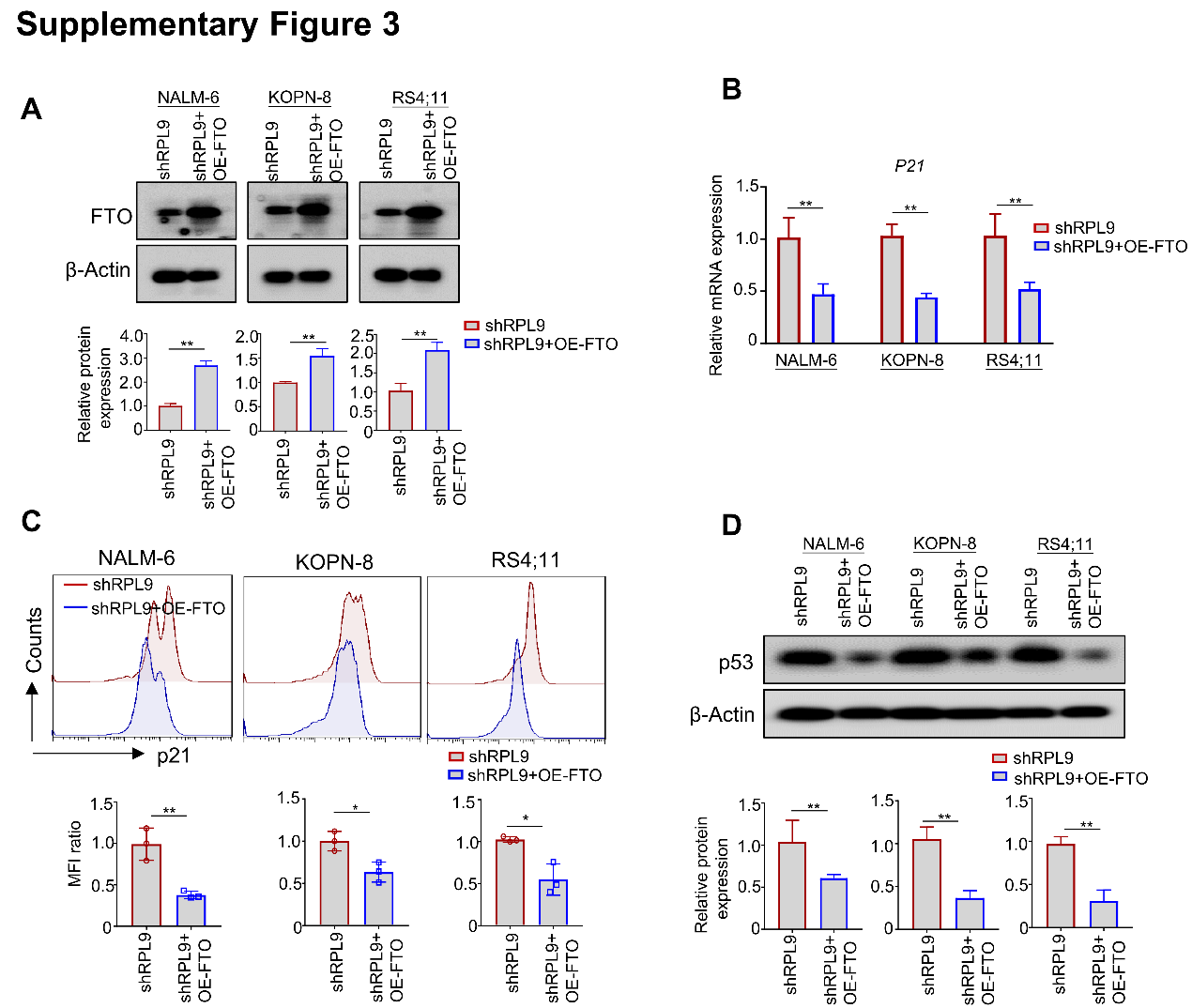
**

**Supplementary Figure 3. (A)** NALM-6, KOPN-8 and RS4;11 cells were infected with shRPL9 lentivirus or shRPL9 plus OE-FTO lentivirus, after 72 hours, the protein expression of FTO were detected using immunoblot. **(B)** NALM-6, KOPN-8 and RS4;11 cells were infected with shRPL9 lentivirus or shRPL9 plus OE-FTO lentivirus, after 72 hours, the mRNA expression of p21 were shown by using RT-qPCR. **(C)** NALM-6, KOPN-8 and RS4;11 cells were infected with shRPL9 lentivirus or shRPL9 plus OE-FTO lentivirus, after 72 hours, the protein expression of p21 were shown by using flow cytometry. **(D)** NALM-6, KOPN-8 and RS4;11 cells were infected with shRPL9 lentivirus or shRPL9 plus OE-FTO lentivirus, after 72 hours, the protein expression of p53 were shown through immunoblot. Data represent mean ± SD, *P < 0.05, **P < 0.01.
